# Manganese toxicity suppressing nitrogen-fixing bacteria growth and impairing nitrogen uptake and utilization in sugarcane

**DOI:** 10.3389/fmicb.2025.1548896

**Published:** 2025-04-16

**Authors:** Hanyu Zhu, Junchen Pan, Yanyan Wei, Heyong Lan, Shu Yang, Xiaofeng Li, Xinlian Tang

**Affiliations:** ^1^State Key Laboratory for Conservation and Utilization of Subtropical Agri–bioresources, Guangxi Key Laboratory for Sugarcane Biology, National Demonstration Center for Experimental Plant Science Education, Academy of Sugarcane and Sugar Industry, College of Agriculture, Guangxi University, Nanning, China; ^2^Guangxi South Subtropical Agricultural Sciences Research Institute, Chongzuo, China; ^3^Agricultural Technology Extension Station of Yinhai District, Beihai, China

**Keywords:** manganese toxicity, soil physicochemical properties, microbial community, sugarcane growth, sugarcane development, nitrogen uptake

## Abstract

**Introduction:**

Sugarcane (*Saccharum* spp.) is a crucial crop for sugar and bioethanol production. However, sugarcane grown in the acidic soils of southern China often suffers from severe leaf chlorosis due to excessive soil manganese (Mn). This study investigates the effects of Mn toxicity on the physicochemical properties and microbial communities in sugarcane rhizosphere soil, as well as its impact on sugarcane growth and nitrogen uptake and utilization.

**Methods:**

Soil samples were collected from sugarcane fields with varying levels of Mn toxicity. Physicochemical properties of the rhizosphere soil were analyzed, including soil pH, available nitrogen, and microbial community composition. The impact of Mn toxicity on sugarcane growth was assessed through measurements of plant biomass, leaf chlorosis, and nitrogen uptake efficiency.

**Results:**

Mn toxicity significantly lowered soil pH and altered the soil microbial community structure. Bacterial genera such as *Nocardioides* and *Sinomonas*, which are involved in ureolysis, cellulolysis, and Mn oxidation, were promoted. In contrast, genera like *Nitrospirota*, associated with nitrogen fixation, were inhibited. This disruption hindered the conversion of soil ammonium nitrogen to nitrate nitrogen, reducing soil available nitrogen. Consequently, sugarcane growth and development were suppressed, and nitrogen uptake was limited.

**Discussion:**

The findings highlight the detrimental effects of Mn toxicity on sugarcane cultivation in high-Mn areas. The altered microbial community composition and reduced soil nitrogen availability directly impact sugarcane growth. These results underscore the importance of applying appropriate fertilizers to mitigate Mn toxicity and improve soil fertility in such regions. Future research should focus on developing strategies to enhance soil nitrogen cycling and promote beneficial microbial communities to support sustainable sugarcane production.

## 1 Introduction

Manganese (Mn) is an essential micronutrient for plant growth and development (Shao et al., 2017). During photosynthesis, Mn participates in water splitting in photosystem II, provides electrons for the photoreaction, regulates the photosynthetic electron transport chain, affects the production of ATP and NADPH, and then affects carbon assimilation. In terms of respiration, Mn activates respiratory metabolic enzymes such as the pyruvate dehydrogenase complex, promotes the oxidative decomposition of respiratory substrates to produce energy, and participates in the regulation of respiratory chain electron transport and oxidative phosphorylation. During protein synthesis, Mn is involved in amino acid activation and transport and ribosome related processes to facilitate the progress of protein synthesis. In the process of hormone activation, Mn affects the synthesis, transport and signal transduction of plant hormones, thereby indirectly regulating plant growth and development (Livorness et al., 2017). However excess Mn is toxic to plants (Li et al., 2007). Mn toxicity is particularly pronounced when the soil pH is less than 5.5, which leads to a sharp increase in dissolved Mn^2+^ concentration, resulting in Mn toxicity to plants (Ducic and Polle, 2005). In Guangxi, China, the soil is mainly acidic and has high levels of Mn in the soil, limiting plant growth and development (Chen, 2001). The sugarcane grown in acidic soils of southern China suffers severe leaf chlorosis and has weak seedlings due to the excessive soil Mn (Long, 2020; Zhang et al., 2022).

Sugarcane has a high nitrogen (N) demand during its growth cycle. N nutrition is crucial for soil fertility and plant productivity (De Vries et al., 2022). The N cycle plays a vital role in soil ecosystems, incorporating essential processes including N mineralization, nitrification, denitrification, and N fixation (Xiang, 2024). Nitrate N (NN) and ammonium N (AN) are the main forms of N available for plant growth. However, Mn significantly affects soil N transformation. Previous studies have reported that low Mn levels can boost the activities of certain enzymes in the soil rhizosphere, whereas high levels of Mn tend to suppress the activities of multiple enzymes (Wu et al., 2009; Xu et al., 2018). Deng et al. (2017) proposed that high Mn levels may promote nitrate reduction, facilitating the conversion of nitrate ions into ammonium ions. Similarly, there is a significant positive correlation between soluble Mn and AN and a significant negative correlation between soluble Mn and NN (Pan et al., 2021). Elevated Mn levels can alter the physicochemical properties and microbial community structure, in turn affecting N forms and disrupting N uptake and utilization by sugarcane (Deng et al., 2017).

Current research on the association between Mn and N has primarily focused on the effects of Mn on soil enzyme activity and plant N uptake and metabolism (Wang et al., 2018; Gao et al., 2019). Studies on the effect of soil Mn toxicity on soil N transformation, especially on the role of soil microorganisms in soil N transformation, remain limited. In recent years, emerging research has revealed the complex interactions between soil microorganisms and element cycling in various contaminated environments. For example, in the study by Yu et al. (2025), it was found that microorganisms with N-fixing and nitrate-reducing functions can influence arsenic methylation in the rhizosphere, highlighting the intricate relationship between microbial N transformation and other element-related processes. This indicates that understanding the role of microorganisms in the Mn-N interaction is crucial. Moreover, Yu et al. (2024) in demonstrated that plants can selectively recruit specific microorganisms in root-associated niches to adapt to metalloid-metal pollution. This implies that in the context of Mn-contaminated soil, sugarcane may also recruit specific microorganisms to respond to Mn toxicity, and these recruited microorganisms may play a role in N transformation. In addition, research on phosphate-solubilizing bacteria in mining areas has provided insights into the interaction between microorganisms and element cycling. As reported by Hu et al. (2024), which certain microorganisms can affect the immobilization of uranium and the availability of phosphorus through specific metabolic pathways. This shows that microorganisms have the potential to regulate element cycling in complex soil environments.

Therefore, exploring the effects of Mn toxicity on soil N transformation and sugarcane N uptake and N utilization has significant implications for enhancing sugarcane production in high-Mn soils. In the present study, we conducted pot experiments to investigate the effects of Mn toxicity on the physicochemical properties and microbial community structure of sugarcane rhizosphere soil, in addition to the relationship between Mn toxicity and N uptake and utilization. This research will enhance our understanding of the interaction between Mn and N in acidified soils and provide theoretical support for high-yield sugarcane production in high-Mn areas.

## 2 Materials and methods

### 2.1 Experimental design

#### 2.1.1 Laboratory culture conditions under simulated soil Mn stress

The sugarcane variety used for testing was Zhongzhe 9. The key concentration for the Mn toxicity symptoms in sugarcane was determined to be 328 g·kg^−1^ based on preliminary experiments with indoor culture and pot studies. The experiment included two N levels: N1 (0.14 g·kg^−1^) and N2 (0.28 g·kg^−1^), which were selected based on preliminary experiments and relevant literatures (De Vries et al., 2022). Two Mn levels: −Mn (0 g·kg^−1^) and + Mn (328 g·kg^−1^), resulting in five treatments: blank control (CK), T1 (N1 − Mn), T2 (N1 + Mn), T3 (N2 − Mn), and T4 (N2 + Mn). T1, T2, T3, and T4 included N, and T1 and T3 did not include Mn toxicity, whereas T2 and T4 included Mn toxicity. Each treatment included the addition of 0.2 g superphosphate (P_2_O_5_ 12%) and 0.3 g potassium chloride (K_2_O 60%) per kg of soil. Each experiment was performed in triplicate.

Red soil, collected from the experimental base of the Guangxi University College of Agriculture, China, was used for testing. The basic soil physiochemical properties were as follows: total N (TN) 0.73 g·kg^−1^, total phosphorus (P) 0.87 g·kg^−1^, total potassium (K) 2.54 g·kg^−1^, AN 38.31 mg·kg^−1^, NN 20.95 mg·kg^−1^, alkaline hydrolysable N 74.06 mg·kg^−1^, available P 110.62 mg·kg^−1^, available K 134.78 mg·kg^−1^, exchangeable Mn^2+^ 6.83 mg·kg^−1^, easily reducible Mn^2+^ 27.05 mg·kg^−1^, soil organic matter (SOM) 9.87 g·kg^−1^, and pH 5.18. Soil from the 0–20-cm tillage layer was collected, air dried and passed through an 18-mesh sieve. After evenly mixing, 100.00 g of soil was weighed into a culture bottle, and the soil moisture was adjusted to 40% of the maximum field capacity. The culture bottle was sealed, and 5–6 holes were punched into the cap to minimize soil moisture evaporation while ensuring an aerobic environment. All culture bottles were pre-cultured in an incubator at 25°C in the dark for 1 week. The incubator was not shaking during the experiment. The N fertilizer used for testing was urea (N, 46%), with Mn added in the form of anhydrous Mn sulfate. The P fertilizer used was superphosphate (P_2_O_5,_ 12%), whereas the K fertilizer was K chloride (K_2_O, 60%). Subsequently, the fertilizers were added as described above, soil moisture was adjusted to 60% of maximum field capacity, and culturing was continued. Deionized water was added daily to maintain a relatively constant moisture content.

#### 2.1.2 Pot experiment

The pot experiments were conducted in a glass greenhouse. The pot experiments and laboratory culture experiments shared the same protocol. Each pot was filled with 20.0 kg of air-dried soil that had been passed through a 5 × 5-mm mesh sieve. N, P, and K fertilizers were mixed into the soil once according to the different treatments, and a Mn sulfate solution was added to the soil. Soil moisture was adjusted to 60% of maximum field capacity and balanced for 1 week. Each pot contained two Zhongzhe 9 plants. Cuttings, 2–3 cm in length, were taken from single-node stems and soaked in water to encourage sprouting and rooting. Single-node stems with relatively consistent root and shoot growth were selected for transplanting. After cultivating for 60 days, the sugarcane plants were harvested and sampled for analysis to determine plant height, dry biomass, N uptake and other indicators. Each treatment was performed in triplicate.

Before harvesting, the height of the sugarcane plants was measured, and the entire plant was uprooted. After removing larger soil clods, the rhizosphere soil attached to the root surface was gently brushed off with a brush into a sterile bag. The brush was sterilized by autoclaving at 121°C for 20 min before each use to prevent microbial contamination transmitted through the brush. The collected rhizosphere soil was divided into two parts: one was air-dried and used to measure the basic physicochemical properties of the soil, while the other was stored in a freezer at −80°C to analyze microbial community structure and diversity. Sugarcane plants were divided into roots and shoots and stored separately in sterile bags. After washing with water and wiping dry the samples were sterilized at 105°C and dried in an oven at 60°C to a constant weight. The dry matter content was weighed and recorded and the samples were crushed and sieved for N measurement.

### 2.2 Measurement items and methods

#### 2.2.1 Determination of soil and sugarcane N, K, and P contents

Soil pH (5:1 water-to-soil ratio) was measured using the glass electrode method (Bao, 2000); soil available P was measured using the 0.05 mol/L HCl–0.025 mol·L^−1^ 1/2 H_2_SO_4_ method (Bao, 2000); soil TN was measured using the semi-micro Kjeldahl method (Bao, 2000); available K was measured using the 1 mol·L^−1^ NH_4_OAc extraction–flame photometry method (Bao, 2000); alkaline hydrolyzable N was measured using the alkaline hydrolysis diffusion method (Bao, 2000); soil AN was measured using the 2 mol·L^−1^ KCl extraction–indophenol blue colorimetric method (Bao, 2000); SOM was measured using the potassium dichromate volumetric analysis–external heating method (Bao, 2000); soil exchangeable Mn was measured using the 1 mol·L^−1^ NH_4_OAc extraction–atomic absorption spectrophotometry (AAS) method (Bao, 2000); soil easily reducible Mn was measured using the benzenediol–1 mol·L^−1^ NH_4_OAc extraction–AAS method (Bao, 2000). As previously described (Miao et al., 2019), soil NN was measured using dual-wavelength ultraviolet spectrophotometry.

Sugarcane N content was measured as previously described (Bao, 2000). The plant samples were dried, crushed, and digested with H_2_SO_4_–H_2_O_2_. N content was then measured using an automatic N analyzer, and the following indicators were calculated:Sugarcane dry biomass = shoot dry biomass + root dry biomass.


SugarcaneNuptake amount=Ncontent×drybiomass

$$\begin{array}{l} \mathrm{Sugarcane total}\;N\;\mathrm{uptake amount}=\mathrm{shoot}\;N\;\mathrm{uptake amount}\\ +\mathrm{root}\;N\;\mathrm{uptake amount} \end{array}$$

$$\begin{array}{l} \mathrm{Sugarcane}\;N\;\mathrm{uptake efficiency}=[\mathrm{plant total}\;N\;\mathrm{accumulation}\\ /\mathrm{soil available}\;N\;\left(\mathrm{fertilizer}\;N+\mathrm{soil}\;N\right)\times 100] \end{array}$$

Sugarcane inorganicNcontent=AN content+NNcontent

#### 2.2.2 Soil total DNA extraction, PCR amplification, and sequencing

Total DNA was extracted from the soil samples using the conventional cetyltrimethylammonium bromide method. DNA integrity was determined using agarose gel electrophoresis, and DNA concentration was determined using a NanoDrop device (Gao, 2014). An appropriate amount of DNA extract was placed in a centrifuge tube and diluted to 1 ng·μL^−1^ with sterile water. The diluted genomic DNA was used as a template to synthesize specific barcoded primers according to the designated sequencing region, and PCR amplification was performed using the ABI GeneAmp® 9,700 PCR system (Thermo Fisher Scientific, Waltham, MA, USA). The amplification primers and sequences for the bacterial 16S V3–V4 region were 338F (5′-ACTCCTACGGGAGGCAGCAG-3′) and 806R (5′-GGACTACNNGGGTATCTAAT-3′). The PCR amplification reaction mixture was as follows: 20:4 μL of 5 × FastPfu buffer, 2 μL of 2.5 mmol·L^−1^ dNTPs, 0.8 μL of 5 μmol·L^−1^ forward and reverse primers, 0.4 μL of Fast Pfu polymerase, 1 μL of 20 ng·μL^−1^ template DNA, and 0.2 μL of bovine serum albumin, topped up with sterile water to 20 μL. The PCR reaction procedure was as follows: S1, pre-denaturation at 95°C for 3 min, one cycle; S2, denaturation at 95°C for 30 s, annealing at 55°C for 30 s, extension at 72°C for 30 s, 27 cycles; S3, extension at 72°C for 10 min, one cycle.

PCR products were detected using agarose gel electrophoresis. Equal amounts of the samples were mixed according to the concentrations of the PCR products. The fully mixed PCR products were purified using 1 × TAE 2% agarose gel electrophoresis, excised, and recovered using the Axyprep DNA Gel Recovery Kit (AXYGEN, Union City, CA, USA). Following purification, the samples were sent to Majorbio Bio-Pharm Technology (Shanghai, China), for high-throughput sequencing on the Miseq PE300 platform (Illumina, San Diego, CA, USA).

#### 2.2.3 Bioinformatics analysis and data processing

Quality control was performed on the raw data obtained from sequencing, which involved (1) filtering bases with quality values <20 at the tail-end of reads and removing reads with <50 bp after quality control and reads containing an N-base; (2) splicing paired-end reads to form a sequence based on the overlap relationship between paired-end reads, with a minimum overlap length of 10 bp; (3) permitting a maximum mismatch ratio of 0.2 in the overlap regions of spliced sequences, and screening out non-compliant sequences; (4) distinguishing samples based on the barcodes and primers at both ends of the sequence, and adjusting the sequence direction, with zero allowed barcode mismatches and a maximum of two primer mismatches. Splicing was performed using the FLASH software. A 97% similarity was used as the criterion to cluster the sequences into Operational Taxonomic Unites (OTUs) using UPARSE software; singletons and chimeras were removed during the clustering process. RDP Classifier^1^ was used to perform species taxonomic annotation on each sequence to obtain the species taxonomic information of each OTU.

Statistical analysis was performed on the community composition of the samples at the domain, kingdom, phylum, class, order, family, genus, and species levels. The above analytical operations were all completed on the Majorbio Cloud platform^2^.

### 2.3 Statistical analysis

Data analysis and graph plotting were performed using Excel 2016. Significance testing of differences was performed using IBM SPSS Statistics 24 (IBM Corp., Armonk, NY, USA) and Duncan’s new multiple-range test. Analysis and mapping of microbial communities were conducted using the Majorbio Cloud platform (see footnote 2). Statistical significance was set to *p* < 0.05. The structural equation model (SEM) validation metrics included the comparative fit index (≥ 0.90), goodness of fit index (≥ 0.90), chi-squared test (X2), degrees of freedom (df), X2/df ratio standardizing values (X2/df < 3) and the root mean-square error of approximation (≤ 0.08) (Malaeb et al., 2000; Beaumelle et al., 2020). The dataset comprised n = 36 observations without the need for transformation. The SEM was produced using AMOS (v. 26, SPSS, IBM, Chicago, IL, USA).

## 3 Results

### 3.1 Effects of Mn levels on the physicochemical properties and microbial communities of sugarcane rhizosphere soil

#### 3.1.1 Physicochemical properties

Compared with CK, T1 and T3 increased rhizosphere soil pH values, whereas T2 and T4 decreased rhizosphere soil pH values (Table 1). All treatments had no effect on SOM content. All treatments had increased TN, T3 and T4 showed significant increases. NN levels changed variably among treatments. All treatments had significantly increased AN levels. Specifically, the AN levels of T2 and T4 were significantly higher than those of T1 and T3. Under the same Mn conditions, the AN levels of T1 and T2 were significantly lower than those of T3 and T4. T1 and T3 did not show significant increases in exchangeable Mn, whereas T2 and T4 showed highly significant increases in exchangeable Mn. Furthermore, the exchangeable Mn level of T2 was significantly higher than that of T4.

**Table 1:** Effects of Mn levels on the physicochemical properties of sugarcane rhizosphere soil.

Treatments	pH	SOM/(g·kg^−1^)	TN/(g·kg^−1^)	NN/(mg·kg^−1^)	AN/(mg·kg^−1^)	Exchangeable Mn (mg·kg^−1^)
CK	4.95 ± 0.05^b^	15.27 ± 0.51^a^	1.15 ± 0.04^c^	44.10 ± 7.27^c^	42.16 ± 19.59^d^	6.29 ± 1.52^c^
T1	5.04 ± 0.08^b^	14.48 ± 0.15^bc^	1.23 ± 0.02^c^	100.84 ± 4.73^b^	119.34 ± 12.57^c^	9.24 ± 2.01^c^
T2	4.67 ± 0.13^c^	14.55 ± 0.28^b^	1.35 ± 0.07^b^	43.90 ± 2.86^c^	243.64 ± 22.93^b^	321.83 ± 6.49^a^
T3	5.33 ± 0.07^a^	13.85 ± 0.42^c^	1.40 ± 0.08^ab^	167.75 ± 15.48^a^	217.24 ± 20.54^b^	12.52 ± 2.72^c^
T4	4.57 ± 0.02^c^	14.40 ± 0.24^bc^	1.47 ± 0.03^a^	54.19 ± 6.68^c^	357.58 ± 10.11^a^	225.28 ± 11.43^b^

Data are expressed as the mean ± standard deviation. Different letters indicate significant differences (*p* < 0.05).

#### 3.1.2 Bacterial communities

##### 3.1.2.1 Bacterial diversity

**Figure 1:**
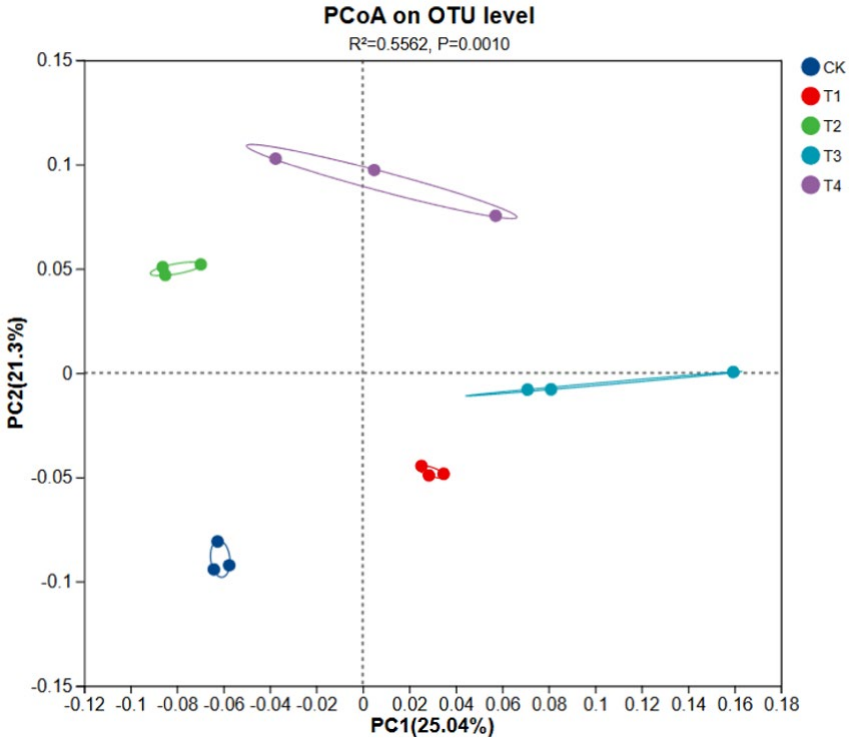
Principal coordinate analysis of soil bacterial community composition: investigating the differences among different treatments.

The library coverage of the five samples ranged between 97.7 and 97.8%, indicating that all bacterial sequences in the soil samples under the five treatments were detectable. In Figure 1, the bacterial Shannon dilution curves for all soil samples across the five treatments plateaued. This suggests that the sequencing data is reliable and accurately represents the overall information of the study samples. The two N levels with or without Mn toxicity showed no significant differences concerning the Shannon, Simpson, Chao1, or ACE (Table 2). This suggests that Mn toxicity does not significantly impact the bacterial diversity of sugarcane rhizosphere soil.

**Table 2:** Effects of Mn levels on the operational taxonomic unit number and alpha diversity indices of soil bacterial communities.

Treatments	Shannon	Simpson	ACE	Chao1	Coverage
CK	5.92 ± 0.06^a^	0.011 ± 0.001^c^	3079.33 ± 14.13^a^	3071.14 ± 45.07^a^	0.977 ± 0.000^b^
T1	5.80 ± 0.01^ab^	0.015 ± 0.000^bc^	2997.63 ± 18.91^ab^	2990.35 ± 70.73^ab^	0.977 ± 0.000^ab^
T2	5.781 ± 0.03^abc^	0.015 ± 0.000^bc^	3078.76 ± 23.91^a^	3077.66 ± 67.30^a^	0.977 ± 0.000^b^
T3	5.600 ± 0.24^bc^	0.024 ± 0.009^a^	2942.50 ± 117.46^b^	2945.80 ± 127.00^ab^	0.978 ± 0.001^a^
T4	5.568 ± 0.06^c^	0.021 ± 0.006^ab^	2920.57 ± 50.07^b^	2908.98 ± 28.67^b^	0.978 ± 0.001^a^

Data are expressed as the mean ± standard deviation. Different letters indicate significant differences (*p* < 0.05).

##### 3.1.2.2 Principal coordinate analysis of bacterial communities under different Mn treatments

The soil bacterial communities of CK and the four treatments showed significant separation (*R^2^* = 0.5562 and *p* = 0.0010; Figure 1), with marked differences between T1 and T2 and between T3 and T4 on the PC1 axis. These results suggest significant differences between the bacterial communities of rhizosphere soil with and without Mn toxicity, regardless of N levels. However, the distribution differences of T1 and T3 on the PC2 axis suggest that N levels affect bacterial communities.

##### 3.1.2.3 Bacterial community composition

**Figure 2:**
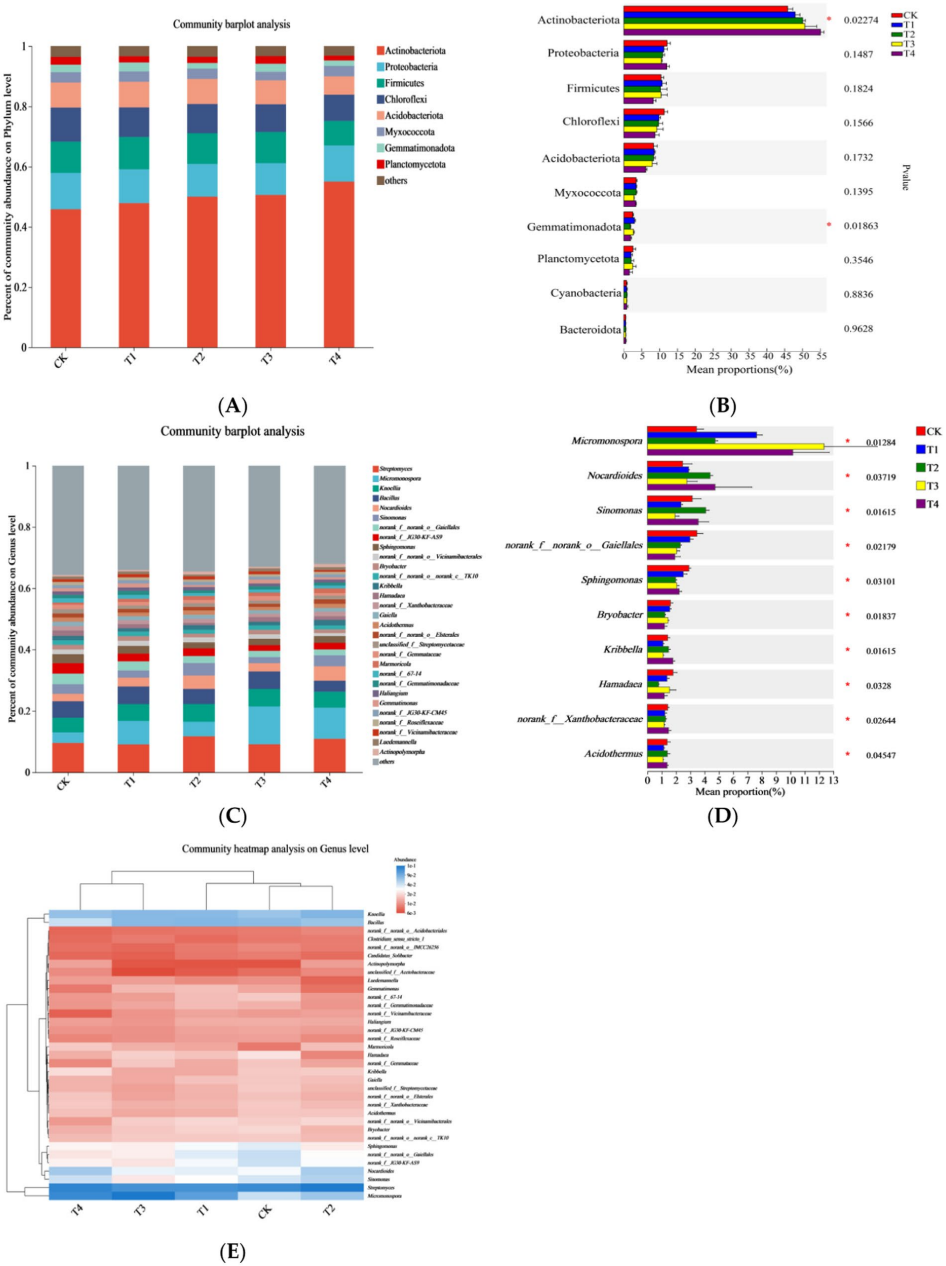
Bacterial community composition under different treatments: displaying the composition characteristics of bacterial communities at the genus level in sugarcane rhizosphere soil under different treatments: **(A)** Bacterial community composition at the phylum level in soil, **(B)** Differences in bacterial taxa at the phylum level in soil, **(C)** Bacterial community composition at the genus level in soil, **(D)** Differences in bacterial taxa at the genus level in soil, **(E)** Heat map of bacterial distribution at the genus level in soil. * Means significant at *p* < 0.05.

Eight phyla with relative abundances exceeding 1% were identified, namely Actinobacteriota, Proteobacteria, Firmicutes, Chloroflexi, Acidobacteriota, Myxococcota, Gemmatimonadota, and Planctomycetota (Figure 2A). The combined relative abundance of these bacteria exceeded 80% across all treatments, suggesting that Mn toxicity did not affect the dominant bacterial taxa in the soil at the phylum level. However, Mn toxicity significantly increased the relative abundance of Actinobacteriota, and decreased that of Gemmatimonadota in sugarcane rhizosphere soil (Figure 2B), changing the relative abundances within the bacterial community. One-way ANOVA followed by Duncan’s *post hoc* test confirmed these changes (*p* < 0.05).

The dominant bacterial genera in the rhizosphere soil were mostly consistent across all treatments and primarily included *Streptomyces*, *Micromonospora*, *Knoellia*, *Bacillus*, and *Nocardioides*. Bacterial taxa at the genus level with relative abundances of >1% were plotted as bar charts (Figure 2C). A total of 28 genera were identified, with the top 10 concerning relative abundance being *Streptomyces*, *Micromonospora*, *Knoellia*, *Bacillus*, *Nocardioides*, *Sinomonas*, the order Gaiellales, unclassified genus, *norank_o_Gaiellalesnorank_f_JG30-KF-AS9*, *Sphingomonas*, and *norank_f_norank_o_Vicinamibacterales*. Mn toxicity did not alter the dominant bacterial taxa at the genus level; however, it significantly affected bacterial relative abundances at the genus level. Specifically, Mn toxicity significantly increased the relative abundances of *Nocardioides* and *Sinomonas*, but decreased those of *Micromonospora* and norank_f_norank_o_Gaiellales (*p* < 0.05) (Figure 2D).

The top 35 bacterial genera were selected based on relative abundance data for each genus in each treatment to investigate the effect of Mn toxicity on the dominant bacterial genera. These top 35 bacterial genera achieved an accumulated relative abundance exceeding 80% across all treatments, thus they are more likely to show meaningful responses to the treatments. A cluster heatmap was plotted using soil treatment and bacterial genus (Figure 2E). In addition, cluster analysis of the different treatments revealed that the similarity of dominant bacterial genera was higher between T2 and T4 and between T1 and T3, suggesting that Mn toxicity altered the relative abundances of dominant bacterial genera in the soil.

##### 3.1.2.4 Soil bacterial functions

**Figure 3:**
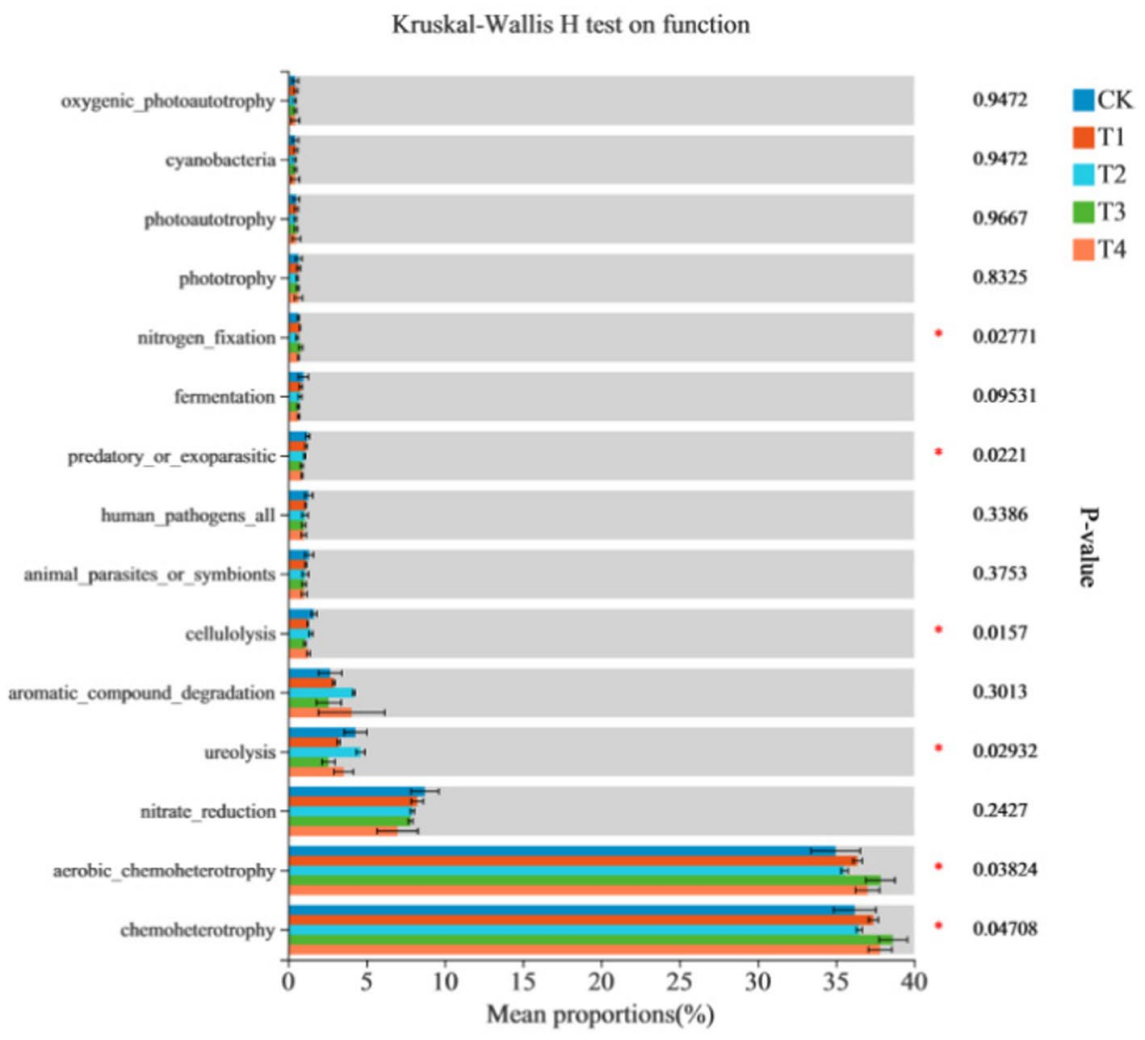
Community heatmap analysis at the genus level: presenting the impact of Mn toxicity on bacterial community. * Means significant at *p* < 0.05.

Based on the taxonomic annotation results of 16S sequences, the FAPROTAX tool was used to annotate the functions of the bacterial communities, which yielded 51 functional groups. The bacterial functional groups with high relative abundances were approximately equal across different treatments (Table 3). However, the remaining functional groups related to the N cycle, such as nitrate respiration, nitrite denitrification, nitrate ammonification, and nitrification, were relatively low, accounting for only 0.004–0.09%. Among the top 15 functional groups ranked by relative abundance, Mn toxicity significantly increased the relative abundances of bacteria functionally annotated as ureolysis, cellulolysis, and Mn oxidation. However, Mn toxicity decreased the relative abundances of bacteria functionally annotated as chemoheterotrophy, aerobic chemoheterotrophy, and N fixation (Figure 3).

**Table 3:** Top 15 bacterial functional groups ranked by soil relative abundance under different Mn toxicity based on FAPROTAX prediction.

Functional groups	CK (%)	T1 (%)	T2 (%)	T3 (%)	T4 (%)
Chemoheterotrophy	36.2 ± 1.4	37.4 ± 0.3	36.5 ± 0.2	38.7 ± 1.0	37.8 ± 0.8
Aerobic chemoheterotrophy	35.0 ± 1.6	36.4 ± 0.3	35.6 ± 0.2	37.9 ± 1.0	37.0 ± 0.8
Nitrate reduction	8.8 ± 0.9	8.3 ± 0.4	8.0 ± 0.1	7.8 ± 0.1	7.0 ± 1.3
Ureolysis	4.3 ± 0.7	3.2 ± 0.1	4.6 ± 0.3	2.6 ± 0.4	3.6 ± 0.6
Aromatic compound degradation	2.7 ± 0.7	2.9 ± 0.1	4.2 ± 0.1	2.6 ± 0.8	4.1 ± 2.1
Cellulolysis	1.7 ± 0.2	1.3 ± 0.0	1.5 ± 0.1	1.1 ± 0.1	1.3 ± 0.1
Animal parasites or symbionts	1.4 ± 0.3	1.2 ± 0.1	1.1 ± 0.2	1.0 ± 0.2	1.0 ± 0.2
Human pathogens all	1.3 ± 0.3	1.1 ± 0.0	1.1 ± 0.2	1.0 ± 0.1	1.0 ± 0.2
Predatory or exoparasitic	1.3 ± 0.1	1.2 ± 0.1	1.1 ± 0.1	0.9 ± 0.1	0.9 ± 0.0
Fermentation	1.0 ± 0.3	0.8 ± 0.1	0.8 ± 0.1	0.7 ± 0.1	0.7 ± 0.1
Nitrogen fixation	0.7 ± 0.1	0.8 ± 0.0	0.6 ± 0.1	0.8 ± 0.1	0.7 ± 0.0
Phototrophy	0.7 ± 0.2	0.7 ± 0.1	0.6 ± 0.1	0.6 ± 0.1	0.7 ± 0.3
Photoautotrophy	0.5 ± 0.2	0.5 ± 0.1	0.5 ± 0.1	0.5 ± 0.1	0.5 ± 0.3
Cyanobacteria	0.5 ± 0.2	0.5 ± 0.1	0.4 ± 0.1	0.5 ± 0.1	0.5 ± 0.3
Oxygenic photoautotrophy	0.5 ± 0.2	0.5 ± 0.1	0.4 ± 0.1	0.5 ± 0.1	0.5 ± 0.3

### 3.2 Effects of Mn toxicity on sugarcane growth, development, and N uptake, and N utilization

#### 3.2.1 Sugarcane growth and development

**Figure 4:**
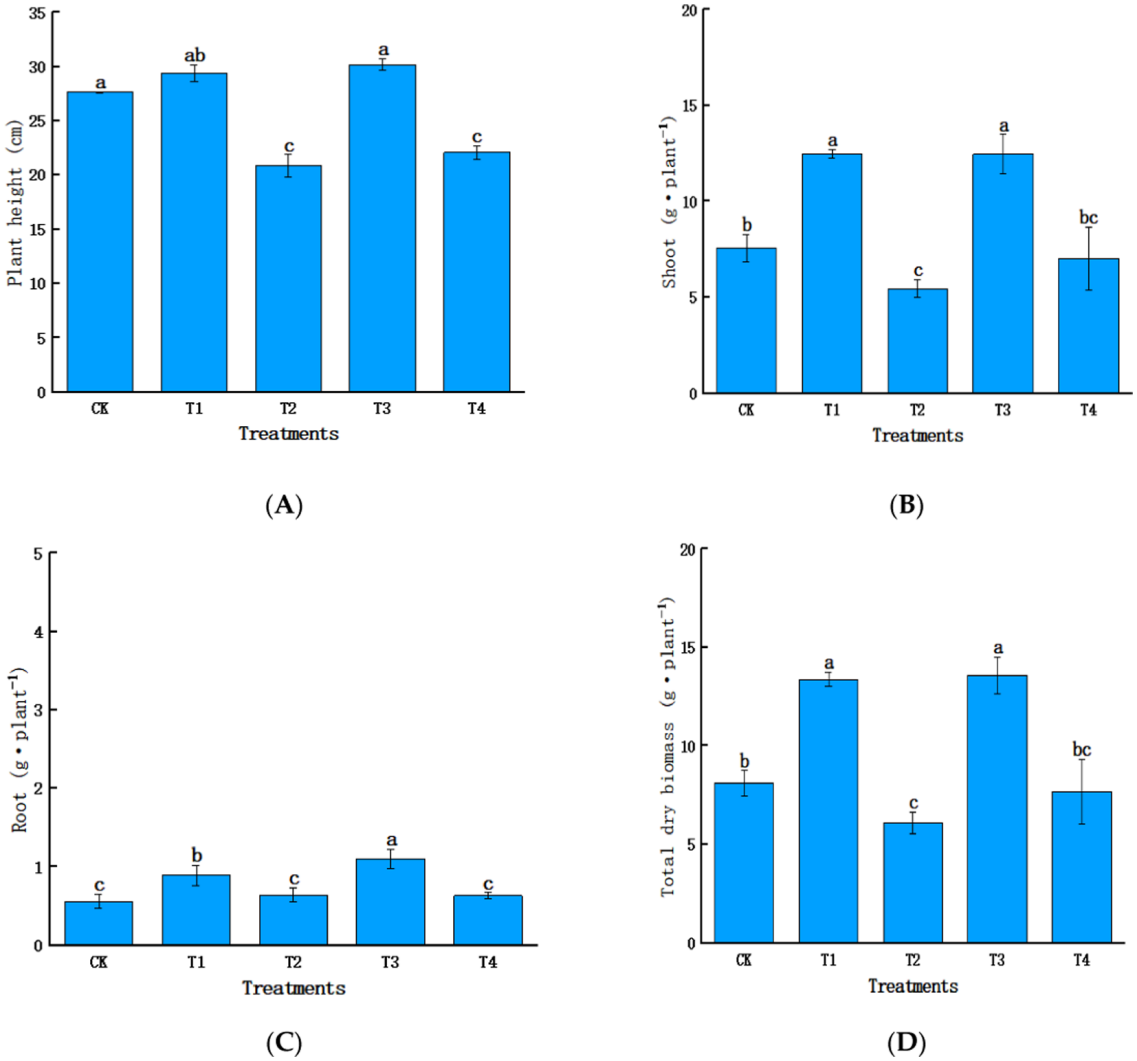
Effects of manganese on the plant height and dry biomass of sugarcane: a comparison among different treatments: **(A)** Plant height, **(B)** dry biomass (shoot), **(C)** dry biomass (root), and **(D)** dry biomass (total dry biomass). Different letters indicate significant differences (*p* < 0.05).

Compared with CK, T2 and T4 showed significant decreases in sugarcane plant height and dry biomass, whereas T1 and T3 showed significant increases in sugarcane plant height and dry biomass (Figure 4). These findings indicate that Mn significantly reduces dry biomass and inhibits sugarcane growth.

#### 3.2.2 Sugarcane N uptake and utilization

**Figure 5:**
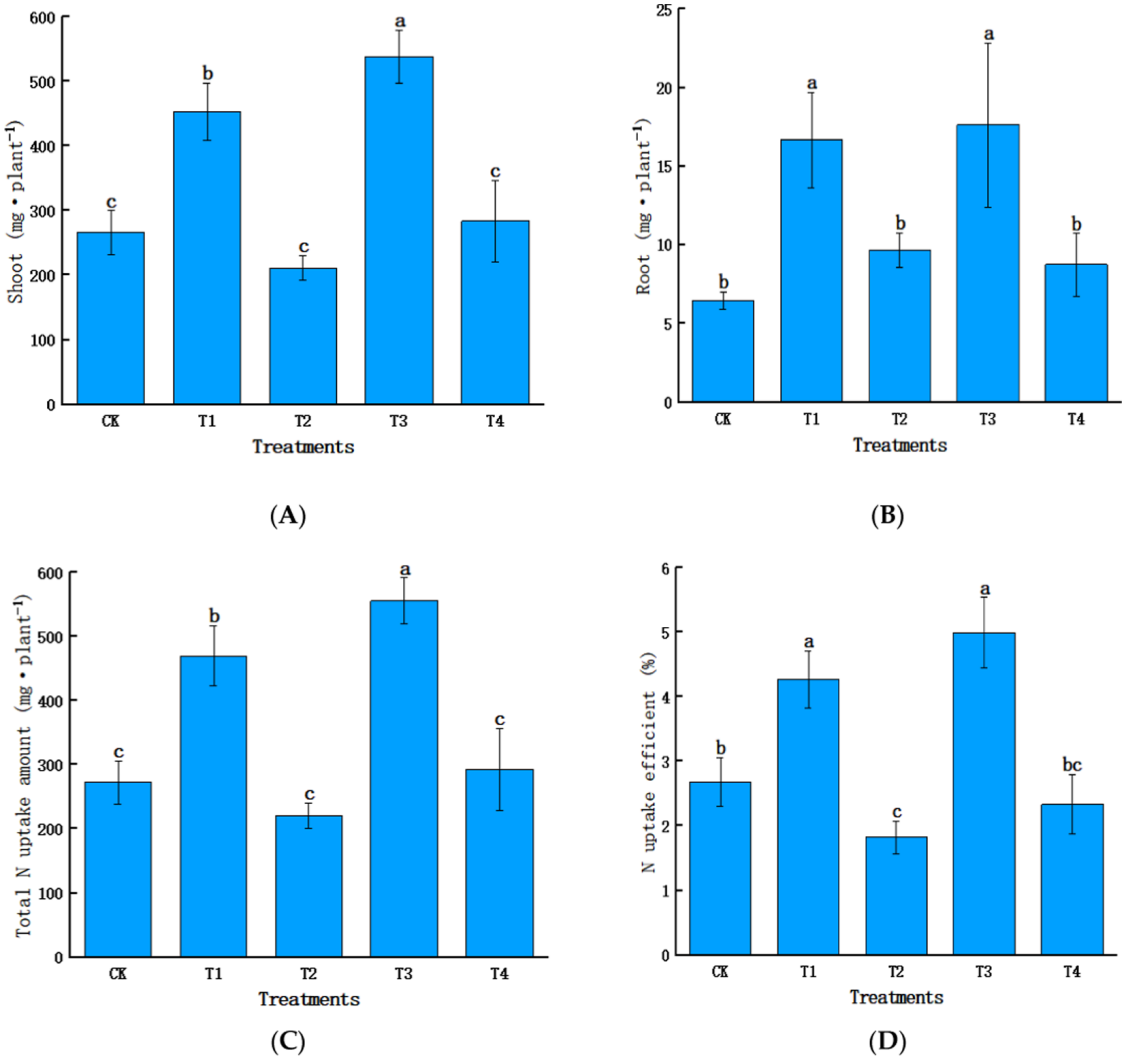
Effects of manganese toxicity on nitrogen uptake and utilization in sugarcane: analysis of the differences in nitrogen uptake amounts and utilization efficiencies under various treatments: **(A)** N uptake amount (shoot), **(B)** N uptake amount (root), **(C)** N uptake amount (total N uptake amount), and **(D)** N utilization efficiency. Different letters indicate significant differences (*p* < 0.05).

T2 and T4 showed significantly lower N uptake amounts than T1 and T3; shoot, root, and total N uptake amounts and efficiency were 53.4, 42.0, 53.0, and 57.4% lower in T2 than in T1, respectively, and 47.3, 50.4, 47.4, and 53.3% lower in T4 than in T3, respectively (Figure 5). This indicates Mn toxicity significantly inhibits N uptake and accumulation and reduces N uptake and utilization efficiency in sugarcane.

### 3.3 Correlation analysis of soil physicochemical properties and sugarcane N utilization efficiency with bacterial community structure and diversity

**Figure 6:**
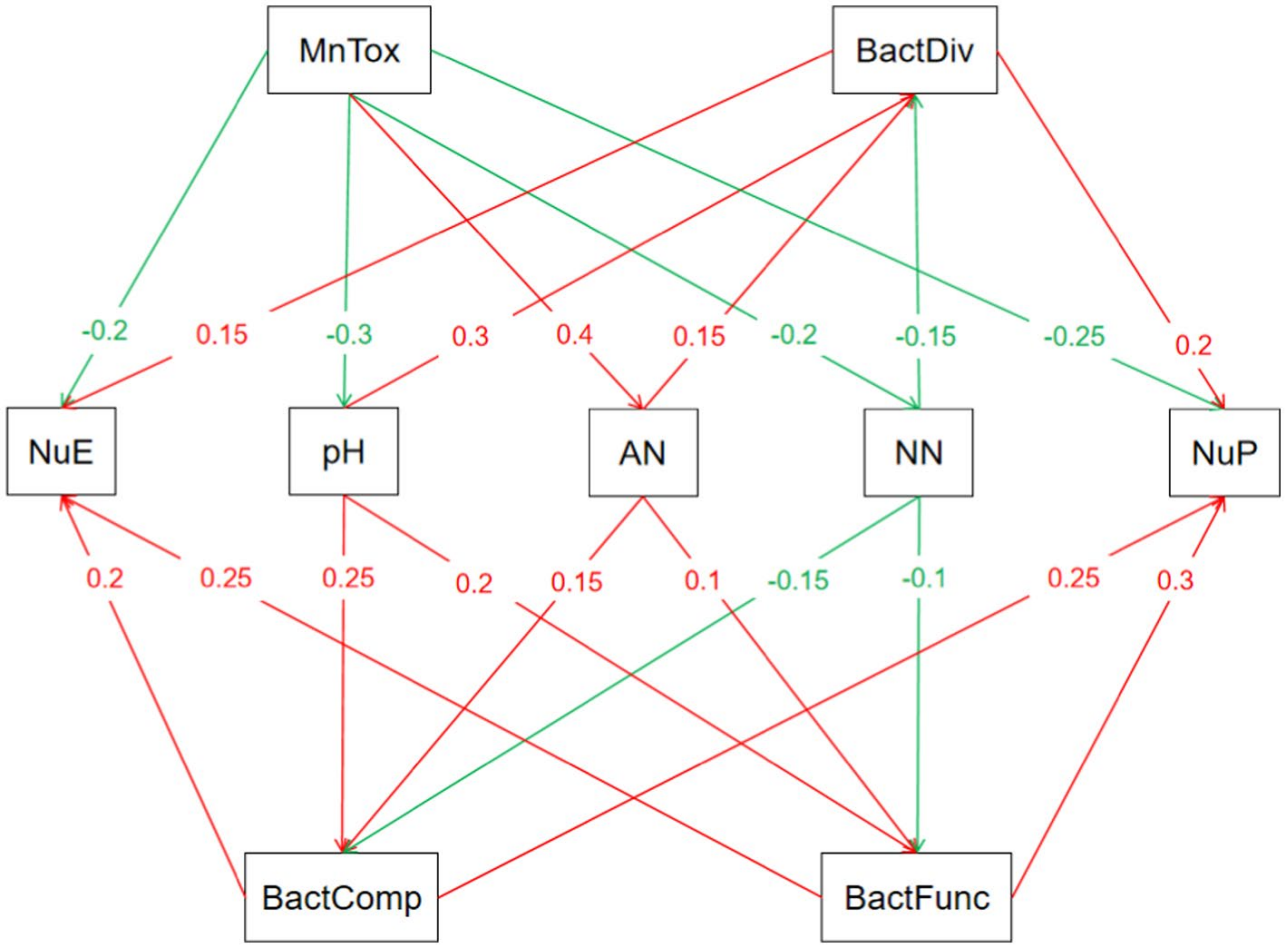
Structural equation modeling of nitrogen uptake and utilization of sugarcane by Mn toxicity: construction and analysis.

The SEM model illustrated the complex mechanism of Mn toxicity on N uptake and utilization by sugarcane. Mn toxicity directly affects soil physical and chemical properties, such as by reducing soil pH and changing soil N forms (increasing AN and reducing NN), and changes in soil physical and chemical properties further affect soil microbial communities, including bacterial diversity, community composition, and function. For example, soil pH has positive effects on bacterial diversity to varying degrees, and changes in soil AN and NN also affect bacterial community structure and function (Figure 6).

**Figure 7:**
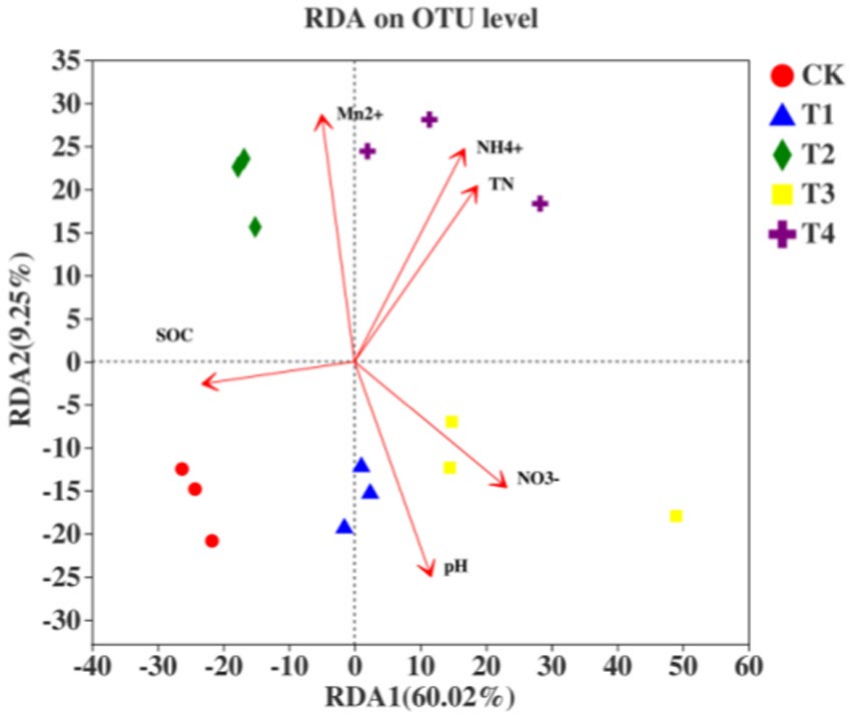
Redundancy analysis of soil bacterial communities and physicochemical properties: revealing the interrelationships under Mn toxicity.

Redundancy analysis demonstrated that soil physicochemical properties significantly affected soil bacterial communities, although the effects varied with different physicochemical factors: soil exchangeable Mn > soil pH > soil AN > soil NN > soil TN > soil organic carbon (SOC) (Figure 7). Soil TN (*R^2^* = 0.7717, *p* = 0.002), NN (*R^2^* = 0.6692, *p* = 0.004), AN (*R^2^* = 0.9253, *p* = 0.001), exchangeable Mn (*R^2^* = 0.8965, *p* = 0.002), and pH (*R^2^* = 0.7707, *p* = 0.001) had highly significant effects on the bacterial community structure (*p* < 0.01). In contrast, SOC (*R^2^* = 0.4682, *p* = 0.027) had a significant effect on the soil bacterial community structure (*p* < 0.05). The soil bacterial communities under T2 and T4 were positively correlated with exchangeable Mn, AN, and TN, whereas those under T1 and T3 were positively correlated with soil pH and NN. These correlations highlight how Mn-induced alterations in soil properties affect the abundances of specific bacterial genera, which in turn influence the N uptake capacity of sugarcane.

**Figure 8:**
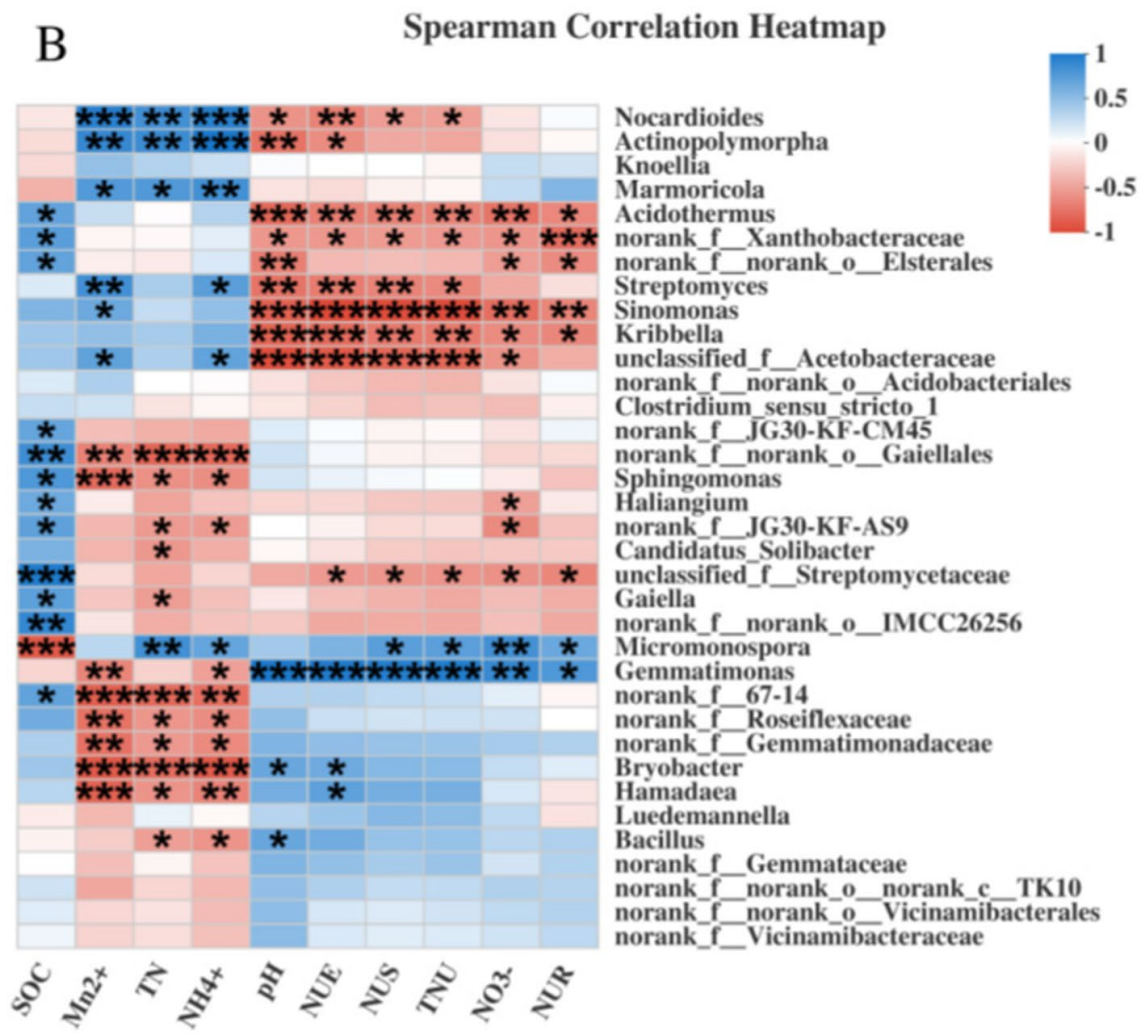
Correlation analysis of genus—level bacteria with rhizosphere soil physicochemical properties and sugarcane N uptake and utilization efficiency under Mn Toxicity. * Means significant at *p* ≤ 0.05, ** means significant at *p* ≤ 0.01, *** means significant at *p* ≤ 0.001.

In terms of microbes at the genus level, there were notable correlations with soil properties and sugarcane N uptake. Soil pH was negatively correlated with *Nocardioides* and *Sinomonas*, whereas SOC exhibited positive and negative correlations with norank_f_norank_o_Gaiellales and *Micromonospora*, respectively. TN, NN, AN, and exchangeable Mn also showed significant correlations with various genera. These relationships indicated that changes in soil properties due to Mn toxicity affected the abundances of various bacterial genera. For example, the increase in exchangeable Mn promoted the growth of *Sinomonas* and *Nocardioides* but inhibited that of norank_f_norank_o_Gaiellales. With regard to sugarcane N uptake, *Micromonospora* was positively correlated with root and shoot N uptake, whereas *Sinomonas* and *Nocardioides* showed negative correlations. These correlations demonstrated how Mn-induced changes in the bacterial community influenced the ability of sugarcane to take up and utilize N, thus highlighting the complex interactions between soil microbes, soil properties, and sugarcane N dynamics (Figure 8).

## 4 Discussion

### 4.1 Effects of Mn toxicity on the physicochemical properties and microbial communities of rhizosphere soil

These findings indicate that Mn toxicity does not significantly affect SOM, significantly decreases soil pH, inhibits the conversion of soil AN to NN, attenuates nitrification, increases soil AN, or increases exchangeable Mn. These results are consistent with previous findings (Deng et al., 2017; Pan et al., 2021), suggesting that Mn toxicity can severely reduce soil nitrification and suppress the conversion of soil AN to NN, thereby enhancing soil AN. We infer that low soil NN occurred because the large increase in Mn increased the amount of Mn oxide (Gu et al., 2020), exerting a strong toxic effect on microbes. Furthermore, since oxidization and release of H^+^ under Mn^2+^ stress is enhanced, the decrease in soil pH lowered microbial activity (Killham, 1990). Such factors suppress microbial activity related to nitrification, in turn elevating soil AN and significantly inhibiting soil NN.

The findings of this study suggest that different N application levels influence bacterial diversity in the absence of Mn toxicity. High N application significantly increased the abundance of Actinobacteria and Firmicutes and markedly decreased N-cycling phyla, such as Acidobacteria, Verrucomicrobia, Cyanobacteria, and Planctomycetes (Ramirez et al., 2012; Yang et al., 2021). However, Mn toxicity did not significantly alter bacterial community diversity in sugarcane rhizosphere soil. The *α* diversity analysis, including Shannon index (5.568–5.92), Simpson index (0.011–0.024), Chao1 index, and ACE index (Table 2), indicated that Mn treatment did not significantly change the overall diversity of rhizosphere soil bacterial community (*p* > 0.05); however, it significantly altered the functional structure of the community through selective enrichment of Mn-oxidizing bacteria (such as *Sinomonas*) and inhibition of *Nitrospirota* (Figure 2D and Figure 3). Principal coordinate and cluster analyses indicated that Mn toxicity significantly altered the relative abundances of each bacterial community. Specifically, Mn toxicity significantly increased the relative abundances of bacterial functional groups involved in ureolysis, cellulolysis, and Mn oxidation and decreased the relative abundances of those involved in chemoheterotrophy, aerobic chemoheterotrophy, and N fixation. Mn toxicity can trigger bacterial stress, resulting in the production of additional Mn oxidation enzymes, stress-resistant proteins, and other compounds. Consequently, this process boosts the population of bacteria associated with Mn oxidation (Sun et al., 2019). Oxidative stress led to cell damage and disrupted metabolism, resulting in a reduced abundance of chemoheterotrophic groups with Mn stress (Sun et al., 2019). Aerobic chemoheterotrophy groups decreased because of interference of enzyme activity and cell death (Wang et al., 2014). Mn stress inhibited N accumulation and nodule fixation, decreasing the abundance of N fixation groups (Jin et al., 2015). Acidobacteria and its group branches are the primary marker species responding to Mn toxicity, and their relative abundance is significantly positively correlated with the exchangeable Mn content in the soil (Li et al., 2023).

Additionally, Actinobacteriota, which proliferates under Mn stress, can mitigate Mn toxicity through its metabolic processes (Kamika and Momba, 2013; Bier et al., 2015). Conversely, the relative abundance of Gemmatimonadota and other phyla declined under Mn stress, demonstrating their susceptibility to heavy-metal toxicity (Li et al., 2021). At the genus level, Mn toxicity significantly increased the relative abundances of *Nocardioides* and *Sinomonas* and decreased those of *Micromonospora* and norankf_Norank_o_gaiellales. This suggests that different bacterial genera exhibit different sensitivity levels to Mn stress (Martin and Waters, 2022). Numerous low-molecular-weight organic compounds, such as organic acids, are secreted by plant roots in soils with high heavy-metal levels to resist external stress (Ge et al., 2019; Li et al., 2023). Our redundancy analysis showed that soil physicochemical properties significantly impacted bacterial communities. Therefore, Mn toxicity could alter the rhizosphere microenvironment, affecting the structure of rhizosphere bacterial communities.

Furthermore, the mineralization of soil organic N is closely associated with SOM decomposition. The decomposition of macromolecules, such as lignin and cellulose, determines the availability of soil N (Berg, 2014; Castro-Díez et al., 2014). As urea is a common N source, ureolysis is crucial to soil N mineralization. Among the numerous microbes involved in ureolysis and organic matter decomposition, specific taxa, particularly Mn-resistant bacteria are the primary decomposers in ureolysis that can also decompose lignin and cellulose through extracellular hydrolases, promoting soil ammonification (Phillips et al., 2015; Ribbons et al., 2016). Therefore, Mn toxicity may promote the relative abundances of bacteria related to ureolysis and organic matter decomposition (e.g., cellulose and lignin decomposition), enhancing soil N mineralization. However, under N amendment, the promotion of ureolysis by Mn toxicity may be lowered. This may be due to catabolic repression, where the presence of excess N suppresses the expression of genes involved in ureolysis (Kumar et al., 2009). Additionally, N amendment can lead to shifts in soil pH, which may further affect the activity of ureolytic bacteria. For instance, high N levels can lower the soil pH, thus creating a more acidic environment that may inhibit the growth and activity of certain ureolytic bacteria (Vitousek et al., 2010). These mechanisms may interact and collectively result in reduced ureolysis under N amendment conditions. Yang and Norton (2020) showed that most ureC gene sequences for ureolytic bacteria (> 83%) were annotated as Proteobacteria, Actinobacteriota, and Myxococcota. By screening ureolytic bacteria, Wang et al. (2015) found that Actinobacteriota exerted a ureolytic function. In this study, Mn toxicity significantly increased the relative abundance of Actinobacteriota, with significant increases also observed for *Nocardioides* and *Sinomonas*, consistent with the changes in ureolysis and cellulolysis functional groups. Among these, *Nocardioides* can decompose macromolecular organic matter, such as cellulose and lignin (Lian et al., 2017). Furthermore, *Nocardioides* is involved in ureolysis, thereby contributing to the hydrolysis of urea and the release of ammonia, which enhances soil N mineralization (Wang et al., 2015). This is supported by our data showing a significant increase in the relative abundance of *Nocardioides* under Mn toxicity, aligning with the observed increases in ureolysis-related functional groups. Further correlation analysis revealed that soil AN and exchangeable Mn showed a highly significant positive correlation with *Nocardioides*, whereas sugarcane N uptake and utilization showed a significant negative correlation. Based on their increased abundance under Mn toxicity and the functional group changes, we infer that *Nocardioides* and *Sinomonas* exert ureolytic and cellulolytic functions. Mn toxicity promoted the growth of ureolytic and cellulolytic bacteria and accelerated the hydrolysis of urea and organic matter, thereby promoting ammonification. This, in turn, reduced the available N levels to sugarcane, decreasing soil N uptake and utilization. Notably, we observed low relative abundances of soil nitrifying bacteria (e.g., *Nitrospirota*) and related functional groups (e.g., nitrification) under Mn toxicity (*p* < 0.05). Mn toxicity significantly reduced the relative abundances of nitrifying bacteria, whereas it had no significant impact on the nitrification functional group; this necessitates further investigations to determine whether Mn toxicity affects the role of bacteria in soil nitrification. In addition, we could not identify or classify a large proportion of bacterial OTU functions. Therefore, annotating bacterial functions based on the FAPROTAX tool is significantly limited and requires further development and optimization.

### 4.2 Effects of Mn toxicity on sugarcane growth, development, N uptake, and N utilization

This study found that Mn toxicity significantly reduced sugarcane plant height, dry biomass, and root, shoot, and total N uptake and utilization efficiency, highlighting its negative impact on sugarcane growth, development, and N dynamics. AN and NN are the primary forms of N sources obtained by plants from soil (Xu et al., 2012); their levels determine the N supply capacity of soil (Mo et al., 2008). Sugarcane is a dryland crop that generally uses NN as the primary N source; however, when soil NN level is low, sugarcane can take up AN (Mao et al., 2023). Loqué and von Wirén (2004) found that low levels of AN (20–200 μmol·L^−1^) promote plant growth, whereas excessive AN can be toxic to plant cells, leading to leaf chlorosis and, in severe cases, even death. Hence, reduction in soil NN levels and strict regulation of plant AN uptake may be the primary reasons for reduced N uptake in plants.

Furthermore, roots are the main organs for plants to absorb nutrients; their physicochemical properties and morphology seriously affect N absorption by plants. At the molecular level, the absorption of external NN by plants is mainly mediated by nitrate transporters (Fan et al., 2017). Within a certain nitrate concentration range, the amount absorbed by plants increases as nitrate concentration increases (Wang et al., 2012). In our study, Mn toxicity significantly reduced the relative abundances of soil nitrifying bacteria (e.g., *Nitrospirota*) and related functional groups (e.g., nitrification), leading to decreased soil nitrate N levels. This reduction in soil nitrate N directly impacts the amount of N available for plant uptake, thereby affecting plant growth and development. These results highlight the complex interplay between Mn toxicity, soil N forms, and sugarcane N uptake, all of which are intricately linked to the role of roots and nitrate transporters in nutrient absorption.

## 5 Conclusion

Our experimental findings demonstrated that Mn toxicity significantly altered the composition and abundance of soil microbial communities in sugarcane cultivation soil, thus stimulating the proliferation of ureolytic, cellulolytic, and Mn-oxidizing bacterial genera (e.g., *Nocardioides and Sinomonas*) while suppressing genera involved in N fixation (e.g., *Nitrospirota*). Changes in bacterial abundances altered the soil environment, lowered soil pH, inhibited nitrification in sugarcane rhizosphere soil, and reduced the conversion of AN to NN. Mn toxicity reduced the available N levels in soil and suppressed sugarcane growth and development and N uptake and utilization. These findings suggest that when sugarcane is cultivated in areas with high Mn levels, careful monitoring of soil Mn is essential. Strategic implementation of organic amendments (e.g., organic fertilizers) is recommended to ameliorate soil acidification in sugarcane cultivation soil, thereby alleviating Mn toxicity through chelation of excess Mn ions and concurrently enhancing N uptake and utilization in sugarcane. Fertilizer application can be optimized by systematically monitoring critical soil parameters, such as pH and Mn speciation, alongside shifts in microbial communities, to develop nutrient management protocols.

## Data Availability

The raw sequencing data for both the metagenomic datasets and the 16S rRNA amplicon datasets utilized in this study are available at the China National Gene Bank under the accession number PRJNA1237512.

## Footnotes

^1^ http://rdp.cme.msu.edu/

^2^ http://www.i-sanger.com/project/index.html
