# Supplementary figures and images for: Manganese toxicity suppressing nitrogen-fixing bacteria growth and impairing nitrogen uptake and utilization in sugarcane
Source: Front Microbiol. 2025 Apr 16;16:1548896. doi: 10.3389/fmicb.2025.1548896 (PMC12040925; doi:10.3389/fmicb.2025.1548896)

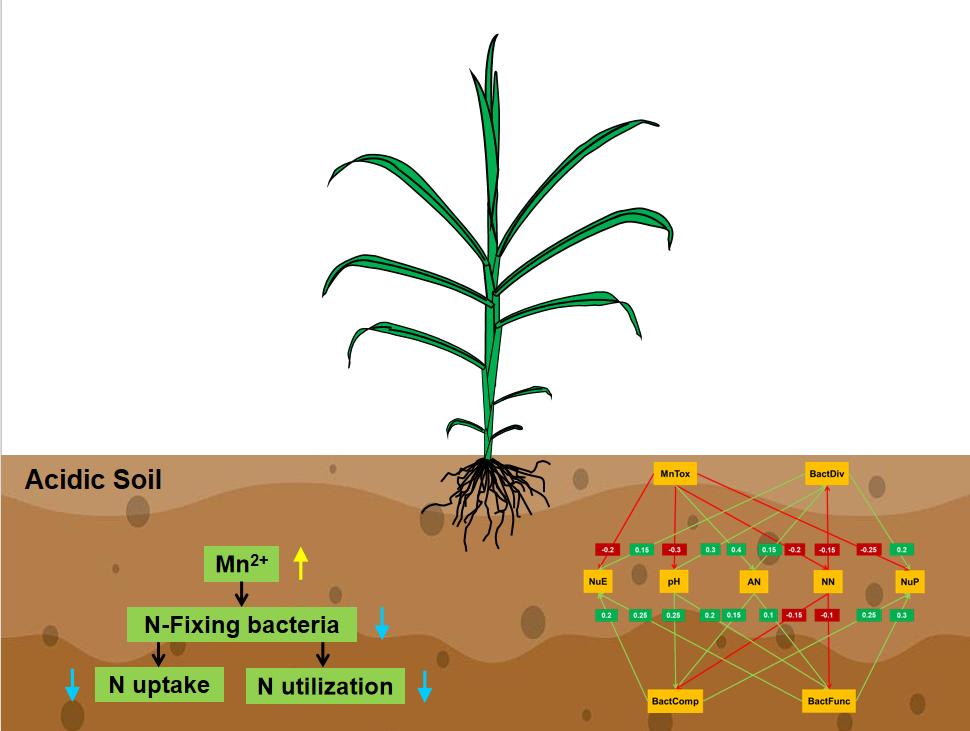

Supplement: Supplementary file 1 [file Image_1.JPEG]
